# Molecular Identification of Sex in* Phoenix dactylifera* Using Inter Simple Sequence Repeat Markers

**DOI:** 10.1155/2016/4530846

**Published:** 2016-06-07

**Authors:** Abdulhafed A. Al-Ameri, Fahad Al-Qurainy, Abdel-Rhman Z. Gaafar, Salim Khan, M. Nadeem

**Affiliations:** Department of Botany and Microbiology, College of Sciences, King Saud University, Riyadh 11451, Saudi Arabia

## Abstract

Early sex identification of Date Palm (*Phoenix dactylifera* L.) at seedling stage is an economically desirable objective, which will significantly increase the profits of seed based cultivation. The utilization of molecular markers at this stage for early and rapid identification of sex is important due to the lack of morphological markers. In this study, a total of two hundred Inter Simple Sequence Repeat (ISSR) primers were screened among male and female Date palm plants to identify putative sex-specific marker, out of which only two primers (IS_A02 and IS_A71) were found to be associated with sex. The primer IS_A02 produced a unique band of size 390 bp and was found clearly in all female plants, while it was absent in all male plants. Contrary to this, the primer IS_A71 produced a unique band of size 380 bp and was clearly found in all male plants, whereas it was absent in all the female plants. Subsequently, these specific fragments were excised, purified, and sequenced for the development of sequence specific markers further in future for the implementation on dioecious Date Palm for sex determination. These markers are efficient, highly reliable, and reproducible for sex identification at the early stage of seedling.

## 1. Introduction

Date Palm (*Phoenix dactylifera* L., Arecaceae family) (2*n* = 36) is a long living dioecious monocotyledon, which is cultivated in arid zones for food, fiber, and shelter [1, 2]. The Arab countries account for 60 percent of the world's production with approximately 800 different kinds of date cultivars [3], which makes Palm tree the major plantation crop in Arab world [4].

The sexual propagation of Date Palm results in seedlings variation which may be different from the mother tree and among them in fruit quality, production potential, and harvesting time. However, the high genetic variability among the offspring may lead to better characteristics for their survival in the natural habitat (e.g., disease resistance) or in their quality [5]. Unfortunately, the sex of individual Date Palm plants cannot be determined until they reach reproductive age, between five and ten years old [6]. This significantly increases the cost of cultivating dates from seeds, as only the female plants bear fruit. A reliable method of early detection of Date Palm sex would significantly increase the profits of seed based cultivation [7].

In the last two decades, there have been serious efforts to understand the basis of sex identification in Date Palm and to develop methods of identifying the sex at an early stage of seedlings. Several types of markers such as biochemical and molecular markers are available for the identification of male and female dioecious crop plants at early stage of development and proved to be beneficial [8]. Inter Simple Sequence Repeat (ISSR) marker is a simple, easy, and quick assay to perform and offers several advantages over other dominant markers and thus has been adopted widely for plant genome analysis [9, 10]. This marker system has rarely been used for sex identification in dioecious plants [11–14].

Until now, the research is limited in the field of sex identification in the germinated seeds of Date Palm cultivars in the Kingdom of Saudi Arabia. There are not enough reproducible markers available for the sex identification in case of Date Palm. Therefore, this research aimed to develop molecular markers for early sex identification at seedling stage through comparative study of male and female Date Palm plants using ISSR and SSR molecular markers.

## 2. Material and Methods

### 2.1. Plant Collection

Leaf samples of ten pairs of different male and female Date Palm cultivars were collected from Al-Rajhi Farm (Al-Qassim, Saudi Arabia) and directly stored at −80°C (Table 1).

### 2.2. Genomic DNA Extraction and PCR Reaction

A modified CTAB (cetyl trimethyl ammonium bromide) procedure based on the protocol of Khan et al. [15] was adopted for obtaining good quality and quantity of genomic DNA. Concentration and purity of extracted DNA were determined by Nanodrop 8000 spectrophotometer. The DNA samples were diluted to a working concentration (20–25 ng/*μ*L) for PCR amplification. Screening of primers was carried out using bulk analysis by pooling genomic DNA from ten male and female plants separately (Table 1).

### 2.3. ISSR Marker Profiling

Two hundred ISSR oligonucleotides (Macrogen Inc., Korea) composed of short tandem repeat sequences with anchor and representing different microsatellites (di- and trirepeats) have been used as generic primers in PCR amplification. PCR was performed in 25 *μ*L reaction using PCR bead (GE healthcare, UK) supplied with all PCR components except primers and DNA. The reaction mixture contained 50 ng DNA and 20 pmol primer for amplification in PCR. The reactions were performed in Applied Biosystems Veriti Thermal Cycler with an initial denaturation step at 94°C for 5 minutes, followed by 40 cycles of 94°C for 1 minute; annealing temperatures were optimized according to primer (Table 2) for 1 min and extension at 72°C for 1.5 minutes. The final extension step was at 72°C for 7 minutes.

### 2.4. Sex-Specific Band Selection and Sequencing

The primers showing unique bands in bulked samples were further used with separate male and female Date Palm samples (seven each). The DNA marker that was present in corresponding male or female samples and absent in the alternate sex samples was recognized as a potential sex-linked marker. The candidate sex-linked markers were then eluted from agarose gel using Wizard SV Gel and PCR Clean-Up System (Promega) according to manufacturer's instructions. Sequencing was carried out using the same primers as applied for unique marker amplification (Macrogen Inc., Korea).

## 3. Results and Discussion

In various economically important plants, such as Date Palm, the female plants produce the dates which have socioeconomic value [16]. Knowledge about the sex of the Date Palm seedlings prior to their transplantation in the field can be of great agronomic potential to make best use of the available resources, especially land usage [17]. In this work, the simple and reproducible ISSR markers were used to identify sex-specific DNA markers to identify date palm sex at seedling stage. Two hundred ISSR primers were used in PCR with genomic DNA of male and female Date Palm. Out of them, two putative sex-linked primers, that is, IS_A02 and IS_A71, gave sex-specific banding pattern (Table 2), while other remaining primers amplified fragments which were not present exclusively either in male or female.

The above low outcome could result from insignificant differences in X and Y chromosome sequences [18] and the limited number of bands generated by ISSR primers [19]. Therefore, without the luck factor, one could test hundreds of primers with no results. Twenty-two ISSR primers were used in* Humulus lupulus* L. and found two male-specific band sequences [11], whereas 80 ISSR primers were used in* Simmondsia chinensis* and found two male-specific band sequences [20].

**Figure 1:**
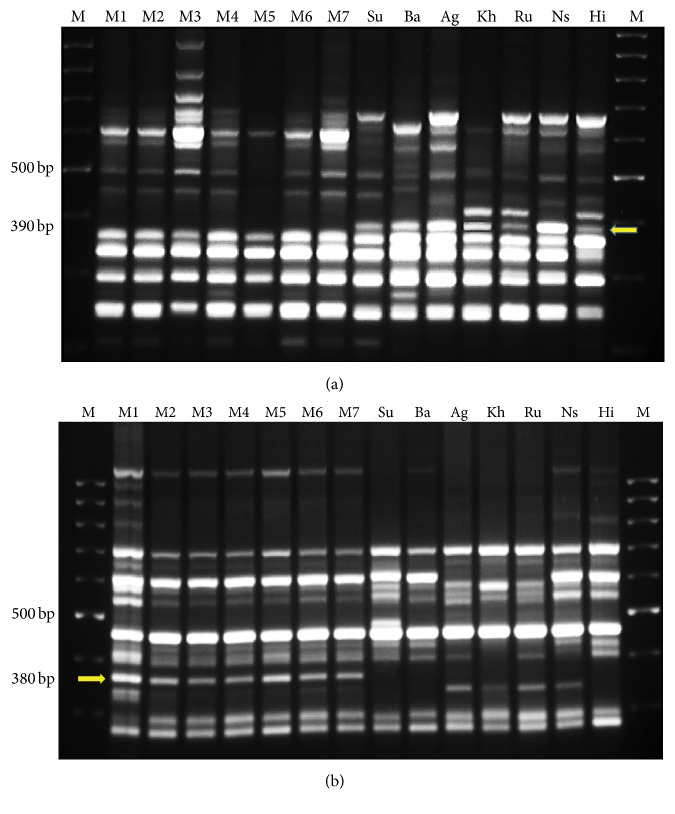
ISSR marker profile for seven pairs of female and male cultivars using the primers (a) IS_A02 and (b) IS_A71. (M: 100 bp DNA ladder).

The primer IS_A02 amplified unique band of size 390 bp in the female, whereas similar fragment was absent in the male in bulk samples. On the contrary, the primer IS_A71 amplified the band of size 380 bp which was unique in the male, while it is consistently absent in the female bulk samples. These results were further validated on seven females (different cultivars) and male plant samples separately and their reproducibility was proven through the same PCR reaction conditions (Figures 1(a)-1(b)).

In this context, only few studies were conducted using ISSR markers to evaluate sex discrimination in dioecious plant species. Sarmah and Sarma [21] screened 30 ISSR primers in* Calamus tenuis* and found only one female-associated marker. Aleksandrov et al. [13] screened 36 primers in* Humulus japonicus*, and found only one male-linked marker. Nanda et al. [12] and Korpelainen et al. [22] used 40 primers and found one male-specific marker in* Trichosanthes dioica* and one female-specific marker in* Pseudocalliergon trifarium*, respectively. Sharma et al. [23] obtained one male-specific DNA fragment after screening a total of 42 primers in* Simmondsia chinensis*.

**Figure 2:**
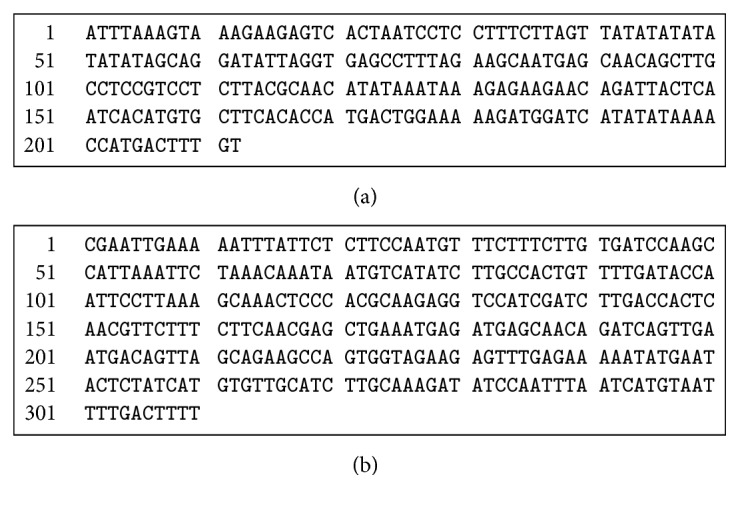
The sequence of (a) the female-specific marker IS_A02 and (b) the male-specific marker IS_A71.

Furthermore, the sequences of the identified putative sex-linked markers were investigated in order to identify the genome segments unique to male or female plant. The generated sequence of putative female-specific fragment of size 212 bp (GC content = 34.4%; A = 79, T = 60, G = 34, and C = 39) (Figure 2(a)) was obtained with primer IS_A02, while the complete generated sequence of putative male-specific fragment of size 310 bp (GC content = 35.2%; A = 101, T = 100, G = 49, and C = 60) was obtained with primer IS_A71 (Figure 2(b)). The generated sequences were submitted at GenBank database of National Centre for Biological Information (NCBI) (Accession Number IS_A02, (KU323794); IS_A71 (KU323795)). These sequences did not show any homology to the other taxa at GenBank database (http://blast.ncbi.nlm.nih.gov/Blast.cgi). These observations were expected due to the usage of random molecular markers (i.e., ISSR) to identify genomic sex linked tags, which are usually from the genomic sites controlling the associated trait and not part of the gene(s) [17].

The outcome of this study proves to be ideal for sex identification especially in experiments where the absence of a PCR product in the sample could be a false negative result due to the lack of DNA in the sample or other obstacles in the PCR process [18]. Therefore, it is preferable to use one or two different sex-linked markers to amplify products of different lengths in males and females in the same reaction. In congruence with the aforementioned method, sex-linked markers were developed in* Ginkgo biloba* by using two different product sizes, one for males at 571 bp and the other for females at 688 bp [24].

## 4. Conclusion

The screened ISSR primers (IS_A02 and IS_A71) in this study gave reproducible results for the discrimination of male and female Date Palm plants. The generated unique bands from male and female plant were sequenced and could be used further for identification of sex at early stage of seedlings of Date Palm. Further, more specific primers can be designed from these generated sequences which could be used for sex identification of Date Palm in a more precise way at seedling stage. Using such DNA markers would help the farmers maintain the sex ratio of Date Palm in plantation, thus saving the time, effort, and cost by avoiding the cultivation of too many male plants in any ongoing Date Palm breeding programs at an early stage.

## Figures and Tables

**Table 1:** Date palm cultivars collected from Al-Rajhi farm (Al-Qassim, Saudi Arabia).

Female cultivars	Abbreviation	Male cultivars	Abbreviation
(1) Sukkari	Su	(1) Male-1	M1
(2) Barhi	Ba	(2) Male-2	M2
(3) Agwa	Ag	(3) Male-3	M3
(4) Khalas	Kh	(4) Male-4	M4
(5) Ruthana	Ru	(5) Male-5	M5
(6) Naboot Seif	Ns	(6) Male-6	M6
(7) Hilaly	Hi	(7) Male-7	M7
(8) Deglet Nour	Dn	(8) Male-8	M8
(9) Medjool	Mg	(9) Male-9	M9
(10) Seqae	Se	(10) Male-10	M10

**Table 2:** Sex-specific ISSR primers and their features.

Oligo	Sequence (5′-3′)	Number of base pairs	Annealing temperature	Unique band (bp)
IS_A02	(GA)_9_C	19	52.3	390
IS_A71	(CA)_8_RG	18	48.9	380
